# A Versatile New Model of Chemically Induced Chronic Colitis Using an Outbred Murine Strain

**DOI:** 10.3389/fmicb.2018.00565

**Published:** 2018-03-27

**Authors:** Monica Barone, Florian Chain, Harry Sokol, Patrizia Brigidi, Luis G. Bermúdez-Humarán, Philippe Langella, Rebeca Martín

**Affiliations:** ^1^Department of Pharmacy and Biotechnology, University of Bologna, Bologna, Italy; ^2^Commensals and Probiotics-Host Interactions Laboratory, Micalis Institute, INRA, AgroParisTech, Université Paris-Saclay, Jouy-en-Josas, France; ^3^Sorbonne University – Université Pierre et Marie Curie, Paris, France; ^4^Avenir Team Gut Microbiota and Immunity, Institut National de la Santé et de la Recherche Médicale, Equipe de Recherche Labélisée 1157, Paris, France; ^5^Department of Gastroenterology, Saint Antoine Hospital, Assistance Publique-Hôpitaux de Paris, UPMC, Paris, France

**Keywords:** DNBS, CD-1 mice, gut inflammation, murine IBD model, colitis

## Abstract

Murine colitis models are crucial tools for understanding intestinal homeostasis and inflammation. However, most current models utilize a highly inbred strain of mice, and often only one sex is employed to limit bias. This targeted approach, which in itself is biased, means that murine genetic diversity and sex-related differences are ignored, making it even more difficult to extend findings to humans, who are highly heterogeneous. Furthermore, most models do not examine the chronic form of colitis, an important fact taking into account the chronic nature of the inflammatory bowel diseases (IBD). Here, we attempted to create a more realistic murine colitis model by addressing these three issues. Using chemically induced chronic colon inflammation in an outbred strain of mice (RjOrl:SWISS [CD-1]), we (i) mimicked the relapsing nature of the disease, (ii) better represented normal genetic variability, and (iii) employed both female and male mice. Colitis was induced by intrarectal administration of dinitrobenzene sulfonic acid (DNBS). After a recovery period and 3 days before the mice were euthanized, colitis was reactivated by a second administration of DNBS. Protocol length was 24 days. Colitis severity was assessed using body mass, macroscopic scores, and histological scores. Myeloperoxidase (MPO) activity, cytokine levels, and lymphocyte populations were also characterized. Our results show that the intrarectal administration of DNBS effectively causes colitis in both female and male CD-1 mice in a dose-dependent manner, as reflected by loss of body mass, macroscopic scores and histological scores. Furthermore, colon cytokine levels and mesenteric lymph node characteristics indicate that this model involves immune system activation. Although some variables were sex-specific, most of the results support including both females and males in the model. Our ultimate goal is to make this model available to researchers for testing candidate anti-inflammatory agents, such as classical or next-generation probiotics; we also aim for the results to be more easily transferrable to human trials.

## Introduction

Murine colitis models are crucial tools for understanding intestinal homeostasis and inflammation (Martin et al., 2017). Their use over recent years has resulted in an exponential growth of knowledge on host–bacteria interactions. The most common *in vivo* models use rodents; they mimic different types of colitis with the aim of testing how the microbiota affects colon inflammation. Models can be placed into one of four categories based on their disease induction method: (i) chemically induced colitis; (ii) bacterially induced colitis; (iii) spontaneous colitis (including congenital and genetically engineered forms); and (iv) adoptive-cell-transfer colitis (Martin et al., 2017). All these models have advantages and disadvantages. For instance, the intrinsic similarities and differences between mice and humans as well as external factors (e.g., living conditions and diet) might influence the ability of murine models to represent disease-related changes that occur in human microbiota (Nguyen et al., 2015).

Here, we focus on chemically induced colitis models, which recreate the morphological, histopathological, and clinical features of human inflammatory bowel diseases (IBD) by orally or intrarectally administering various chemical compounds (Randhawa et al., 2014). For example, colitis can be induced by giving rodents drinking water containing dextran sodium sulfate (DSS) for several days (Wirtz et al., 2007). DSS is toxic to colon epithelial cells and causes the complete loss of the surface epithelium in the intestine (Randhawa et al., 2014). The integrity of the mucosal barrier is therefore affected—large molecules can pass through, provoking colitis (Ni et al., 1996). Colitis can also be induced by rectally injecting a haptenating agent dissolved in ethanol, which allows the agent to pass through the mucosal barrier. The agent is then thought to act upon autologous or microbial proteins in the colon, which makes them immunogenic to the host immune system (Wirtz et al., 2007). The most commonly used haptenating agents are trinitrobenzene sulfonic acid (TNBS) and dinitrobenzene sulfonic acid (DNBS). Both TNBS and DNBS produce isolated points of inflammation and necrosis, as well as self-antigens that provoke immune responses (Elson et al., 2005). Although the models are similar, they are not identical—model functionality may vary, depending on host species identity and genetic background (Mizoguchi and Mizoguchi, 2008; Mizoguchi, 2012). Traditionally, acute protocols are used, in which the DNBS/TNBS injection or DSS period is performed just once and the recovery phase is optional (for some examples, see **Supplementary Figure S1**). However, because inflammation can be chronic, a more realistic model would employ a protocol in which colitis is reactivated at least once, thus mimicking flare-ups and relapses. Colitis development is evaluated using changes in body mass, clinical symptoms (e.g., diarrhea, constipation, and bloody feces), colon morphology, and histological features. Furthermore, because this form of colitis is clearly tied to the immune system, colon cytokine concentrations, lymphocyte levels, and myeloperoxidase (MPO) activity (indicator of neutrophil infiltration that reflects the local immune response) are helpful markers of colitis severity (Wirtz et al., 2007; Martin et al., 2014a).

Researchers use these models to identify and characterize candidate anti-inflammatory agents and test their effects on different IBD or other forms of intestinal mucosal inflammation. Such anti-inflammatory agents include for instance different type of molecules and also microorganisms known as probiotics. Probiotics are “live microorganisms that, when administered in adequate amounts, confer a health benefit on the host” (Hill et al., 2014). At present, thanks to our improved knowledge of the human microbiota, candidate probiotics have been identified from among the dominant members of the gastrointestinal tract (GIT) microbiota found in healthy adults. They are referred to as next-generation probiotics (NGPs) and were originally identified as commensal bacteria species that can reestablish or enhance colonization resistance (Pamer, 2016). However, this definition has been expanded rapidly to include more potential health benefits, overlapping with the emerging concept of live biotherapeutics (O’Toole et al., 2017). NGPs must be shown to be safe for the host; able to survive production, storage, and GIT transit; and elicit a positive host response that confers demonstrable health benefits (Martin et al., 2014b). Since these properties are strain specific, each candidate will have to be tested individually (Pineiro and Stanton, 2007; Hill et al., 2014; Miquel et al., 2015). In the normal sequence of events, these functional analyses involve preliminary *in vitro* testing and then preclinical *in vivo* testing in murine models. The final objective is to perform clinical trials in humans.

There are two main challenges in this process. It is necessary to, first, reproduce the *in vitro* results in the *in vivo* models and, second, reproduce the *in vivo* results in clinical trials. The use of several *in vitro* markers and models has been proposed with the aim of linking *in vitro* results with *in vivo* results. For instance, it was recently suggested that the ratio of anti-inflammatory and pro-inflammatory cytokines (interleukin IL-10 and IL-12, respectively) produced by peripheral blood mononuclear cells (PBMCs) upon *in vitro* exposure to probiotic strains could be a predictor of protective effects *in vivo* in a chemically induced murine colitis model (Foligne et al., 2007). Nevertheless, *in vivo* interactions are much more complex than *in vitro* interactions, and it is difficult to identify the best *in vitro* test for predicting the impact that a candidate anti-inflammatory agent will have *in vivo*. The most widely accepted scientific strategy is to employ a combination of several *in vitro* tests. However, transferring murine results onto a human framework is a separate challenge because success depends upon how well effects in rodents translate into effects in humans. Indeed, past studies found that humans did not experience the beneficial effects of anti-inflammatory agents that were observed in a murine model of colon inflammation mimicking IBD. For example, a *Lactococcus lactis* strain secreting IL-10 was found to decrease DSS-induced colitis by 50%; however, humans treated with a biocontained strain (thyA-/hIL-10+) did not experience beneficial effects in a phase II-A trial (Steidler et al., 2000, 2003, 2009).

Although this discordance in the results obtained in murines vs. humans could be due to their intrinsic differences (Nguyen et al., 2015), it could also be that researchers failed to carefully consider model suitability. At present, most models use an inbred strain of mice (individuals are genetically identical because of extensive inbreeding); furthermore, often only one sex is utilized to limit bias. However, this targeted approach itself introduces bias because it ignores natural genetic diversity and sex-related differences. As a result, it becomes even more difficult to extrapolate any knowledge gleaned from murine models to human populations. Here, our aim is to describe a versatile model of chemically induced chronic colitis that utilizes an outbred strain of mice and both females and males. The ultimate objective is to establish a more realistic model for effectively testing anti-inflammatory agents, for example probiotics; the model should be able to better translate effects in rodents to effects in humans.

## Materials and Methods

### Animals, Experimental Design, and Sampling Procedure

We performed two trials looking at chronic colitis development in RjOrl:SWISS (CD-1) mice (Janvier, Le Genest Saint Isle, France) using C57BL/6JRj (Black-6) mice as control in the first trial. The general characteristics of the two murine strains are described in **Table 1**. The experiment was carried by duplicate in two different periods for each trial. In each period, 5 weeks-old mice were distributed into eight cages based on strain and sex (five mice/cage) and evenly assigned to control or treatment groups (one cage/experimental group). For each trial a total of 40 females and 40 males were used (two cage/experimental group, *n* = 10 mice per group) for a total of 160 mice used in all the study including both trials. Mice were maintained in the animal facilities of the French National Institute of Agricultural Research (IERP, INRA Jouy-en-Josas, France) under specific pathogen free (SPF) conditions at 21°C and housed in cages of 5. They were given food and water *ad libitum* and experienced a 12:12 h light-dark cycle. Before the experiments began, animals were kept under these conditions for 1 week to allow them time to acclimate.

**Table 1:** Murine strains used in this study.

Name	RjOrl:SWISS (CD-1)	C57BL/6JRj (Black-6)
Type	Outbred mouse (guaranteed to display less than 1% inbreeding per generation)	Inbred mouse (guaranteed to display autosomal pair homozygosity)
Distributor and origin	Janvier Labs CSAL (Orleans)—1965	Janvier Labs CSAL (Orleans)—1993
Color and related genotype	Albino mouse—Tyrc/Tyrc	Black mouse, an (a/a) non-agouti MHC: Haplotype H2b
Breeding	Good breeder, strong maternal instinct	Good breeder but difficult to rear due to environmental sensitivity, pup cannibalism


**FIGURE 1:**
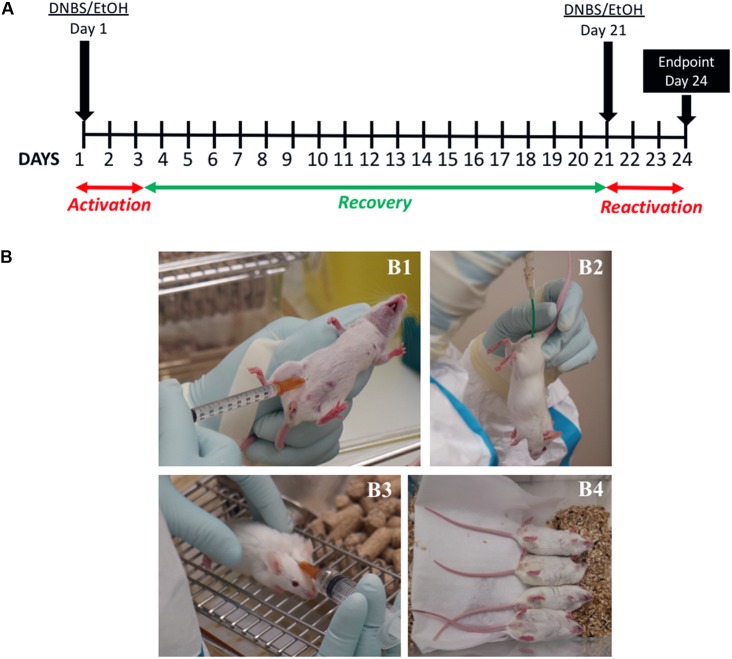
Experimental design **(A)** and key methodological steps **(B)**. Mice were anesthetized with an intraperitoneal (i.p.) injection of ketamine and xylazine **(B1)**. The DNBS solution or the control solution was administered by injection, using a tuberculin syringe and a flexible plastic tube inserted 3.5 cm into the colon **(B2)**. Following the treatment, all mice received a subcutaneous injection of 1 ml of saline solution to prevent dehydration **(B3)**; they were kept in a horizontal position until they awoke **(B4)**.

The experimental protocol for inducing chronic inflammation is illustrated in **Figure 1**. At week 6, mice were anesthetized using an intraperitoneal (i.p.) injection of 0.1% ketamine (Imalgene 1000, Merial, France) and 0.06% xylazine (Rompun, Bayer, France) (**Figure 1B1**). Colitis was induced using DNBS (Sigma-Aldrich, France) resuspended in 50 μl of 30% ethanol (EtOH) in PBS (see **Table 2**). In the first experiment, we wanted to compare inflammation between the two mouse strains; Black-6 is the classical inbred murine strain typically used in these types of experiments. Animals in the treatment group were injected twice with 200 mg/kg of DNBS, which corresponds to 2.7, 3, 4.1, and 4.3 mg/mouse for Black-6 females and males and CD-1 females and males, respectively. In the second experiment, we wished to obtain different degrees of colitis severity in CD-1 mice. We therefore modulated the DNBS dose in the treatment groups. Doses were fixed at 1.5, 2.5, and 3.5 mg/mice, irrespective of mouse mass or sex. In both experiments, the DNBS solution was administered on day 1 by injection with a tuberculin syringe (Terumo, France) and a flexible plastic probe (model V0104040, ECIMED, France) inserted 3.5 cm into the colon (**Figure 1B2**). Control groups were injected with equivalent amounts of the 30% EtOH solution. All mice received a subcutaneous injection of 1 ml of saline solution (0.9% NaCl) to prevent dehydration (**Figure 1B3**). Mice were kept in a horizontal position until they awoke (**Figure 1B4**). These saline injections were repeated daily for the first 3 days (no anesthesia). In this model, colitis develops in the first 3 days following the DNBS injection. During this activation period, the mice lost significant body weight. Mice were allowed to recover for 18 days and then received a second DNBS injection at day 21, reactivating inflammation. During this reactivation period, mice lost weight until the experiment’s endpoint; no saline injections were performed because they could have affected body mass values at the endpoint. Mice were constantly monitored for the duration of the experiment, but especially so during the first 3 days after the DNBS injections. The model we employed in this study is a chronic colitis model because we used two DNBS injections: the first injection induces colitis, a recovery period follows, and then the second injection initiates a reactivation period. Classical acute models utilize a single injection, and colitis induction may or may not be followed by a recovery period. These model types are compared in **Supplementary Figure S1**.

**Table 2:** Dinitrobenzene sulfonic acid (DNBS) doses employed in this study.

Experiments	DNBS Dose
Trial 1: Inflammation patterns in CD-1 mice vs. Black-6 mice	200 mg/kg
	2.7 mg—Black-6 females
	3 mg—Black-6 males
	4.1 mg—CD-1 females
	4.3 mg—CD-1 males
Trial 2: Protocol optimization using CD-1 mice	3.5 mg/2.5 mg/1.5 mg


On day 24, blood samples were collected from the submandibular vein, and mice were euthanized by cervical dislocation. The abdomen was then sterilized with 70% EtOH, the abdominal cavity was opened to collect the spleen and the mesenteric lymph nodes (MLNs), and the entire large intestine was removed. Bowel length was measured, and a small portion of distal colon was immediately placed in a 4% paraformaldehyde (PFA, Prolabo, France) PBS solution for later histological analyses. The intestine was then cut open longitudinally, and the tissue was washed with saline solution after removing the contents. Colon sections of 1 cm were collected and immediately frozen in liquid nitrogen.

All procedures were performed in accordance with European Union (EU) rules on ethical animal care (Directive 2010/63/EU) and were approved by the French Ministry of Research and COMETHEA, the animal ethics committee at INRA Jouy-en-Josas (authorization #3445-2016010615159974).

### Weight Trend and Survival Rate

In both trials, mice were carefully monitored. Their body mass was measured daily throughout the entire experimental period. Saline solution was administered when there was significant loss of body mass to prevent dehydration. In accordance with EU regulations (Directive 2010/63/EU), if mice lost 20% or more of their initial mass and/or showed signs of severe distress, they were euthanized and their id numbers were recorded. Percentage loss of body mass was calculated 3 days after each DNBS injection to compare results among groups.

### Macroscopic Scores

Dinitrobenzene sulfonic acid-induced chronic inflammation is usually visible at the macroscopic level, and inflammation intensity can be evaluated by measuring different parameters, like mucosal damage in colon tissue and stool consistency. In both trials, macroscopic scores were determined using Wallace’s score (Wallace et al., 1989), with the following modifications: tissue sections from each mouse were scored by evaluating ulcerations (score of 0–5), adhesions (presence/absence: 0/1), hyperemia (presence/absence: 0/1), altered transit, such as diarrhea or constipation (presence/absence: 0/1), and increases in colon wall thickness (presence/absence: 0/1; measured using an electronic caliper, Control Company, WVR, United States). The macroscopic scoring system is summarized in **Table 3** and **Supplementary Figure S2**. Although colon length is not typically part of the macroscopic score in these types of models, it was also recorded (see above).

**Table 3:** Macroscopic score.

Characteristic	Score
Ulcers	Absence: 0
	1 ulcer smaller than 0.5 cm: 1
	1 ulcer between 0.5 and 1 cm: 2
	2 ulcers smaller than 1 cm or 1 ulcer larger than 1 cm: 3
	2 ulcers larger than 1 cm: 4
	More than 2 ulcers: 5
Adhesions	Presence: 1; Absence: 0
Hyperaemia	Presence: 1; Absence: 0
Altered transit	Presence: 1; Absence: 0
Colon wall thickening	Presence: 1; Absence: 0


### Histological Scores

In both trials, the tissues collected for the histological analyses were fixed for 24 h in a 4% paraformaldehyde (PFA) solution and then transferred to 70% EtOH. After 24–48 h, the tissues were gradually dehydrated by soaking for 1 h each in 80% EtOH, 90% EtOH, 100% EtOH, and xylene in an automated tissue processer (Leica Biosystem, Germany). Samples were embedded in paraffin using a tissue embedding system (Leica), cut into 5-μm sections using a microtome (UC6, Reicher E - Leica UC6), and then stained with hematoxylin and eosin (HE) for histological scoring using an automated staining system (Leica). All these procedures were performed following conventional methodologies by the histological platform of the GABI Joint Research Unit (INRA, Jouy-en-Josas). Tissues were visualized using a high-capacity digital slide scanner (3DHISTECH Ltd., Budapest) and Panoramic and Case software (3DHISTECH Ltd.). For each animal, at least three tissue sections were evaluated to characterize alterations in mucosal architecture, the degree of immune cell infiltration, and Goblet cell depletion (Ameho score: 0–6) (Ameho et al., 1997).

### Myeloperoxidase Activity and Cytokine Levels

In the second trial, to measure myeloperoxidase (MPO) activity, a 1-cm section of colon tissue from each mouse was weighed and homogenized with Precellys (Bertin Corp., France) in 300 μl of a 0.5% hexadecyltrimethyl-ammonium bromide (HTAB, Sigma-Aldrich) solution in 50 mM potassium phosphate buffer (PPB, pH 6.0); 0.35–0.40 mg of 1.4 and 2.8 mm ceramic beads (Ozyme, France) were added. Each sample was then vortexed for 10 s, centrifuged at 13,000 × *g* and 4°C for 10 min, and then transferred to a 96-well plate. To assay MPO activity, 50 μl of each aliquot was mixed with 200 μl of 50 mM PPB (pH 6.0) containing 0.167 mg/ml of o-dianisidine-dihydrochloride (Sigma-Aldrich, France) and 0.0005% hydrogen peroxide (H_2_O_2_, Sigma-Aldrich). The colorimetric reaction was measured by reading absorbance at 405 nm with a spectrophotometer (Infinite M200, Tecan, Switzerland) at two-time points: immediately and after 1 h. MPO activity was characterized by comparison with a standard (MPO activity of human polymorphonuclear leukocytes, Merck Chemicals, Germany) and then expressed in units/mg of tissue. One activity unit represents the conversion of 1 μM of H_2_O_2_ to water in 1 min at room temperature. To measure cytokine levels, 25 μl of each aliquot or 25 μl of serum were transferred to a 96-well plate. We quantified concentrations of IFN-γ, IL-5, TNF-α, IL-2, IL-6, IL-4, IL-10, IL-9, IL-17A, IL-17F, IL-21, IL-22, and IL-13 using a cytometric bead array system, the Mouse Th Cytokine Panel (13-plex; BioLegend, France), in accordance with manufacturer instructions.

### Lymphocyte Populations in the Spleen and Mesenteric Lymph Nodes

In the second trial, cell suspensions were obtained by mechanically extruding the spleen and MLNs using the plunger end of a syringe and a 75-μm nylon cell strainer (BD, Switzerland). Cells were washed through the strainer using 1 ml of Dulbecco’s Modified Eagle’s Medium (DMEM, Gibco, France) supplemented with 10% fetal bovine serum (FBS, Gibco) and 1% penicillin/streptomycin (PS, Lonza, France). The red blood cell lysing buffer Hybri-Max (Sigma-Aldrich) was used to lyse the erythrocytes present in the cell suspension isolated from spleen, in accordance with manufacturer instructions. For each sample, aliquots of 10^6^ cells were transferred to two 96-well plates (Greiner, France). Following standard protocols, cells were stained with anti-CD4-FITC, anti-CD3e-PE, anti-T-bet-APC, and anti-Gata3-PerCP as well as with anti-CD4-FITC, anti-CD3e-PerCP, and anti-Foxp3-PE, both stainings were performed in the presence of CD16/CD32 (all products came from eBioscience, France) to avoid unspecific staining. In brief, the cells were washed with PBS and incubated for 30 min with 0.5 μg of purified anti-mouse CD16/CD32 and surface antibodies (anti-CD4, anti-CD3) in PBS with 10% FBS and 1% sodium azide (Sigma-Aldrich). Intracellular staining was performed as follows using the Foxp3 Transcription Factor Staining Buffer Kit (eBioscience) in accordance with manufacturer instructions. Briefly, samples were washed with PBS and incubated for 20 min with a permeabilization/fixation buffer. They were then stained with intracellular antibodies (anti-T-bet-APC and anti-Gata3-PerCP or anti-Foxp3-PE) in permeabilization buffer over a period of 30 min. Samples were subsequently washed in permeabilization buffer, resuspended in PBS, and analyzed using an Accuri C6 cytometer (BD). The data obtained from the cytofluorimetric analysis were processed using CFlow Sampler software (BD).

### Gastrointestinal Tract Permeability

In the second trial, 0.6 mg/g of fluorescein isothiocyanate-dextran 4 (FITC-dex 4; Sigma-Aldrich) dissolved in PBS was administered intragastrically to each mouse. Blood samples were collected after 3.5 h as described above, and 80 μl of serum was transferred to a 96-well black plate (Greiner). The concentration of FITC-dex 4 was determined using fluorescence spectrophotometry (excitation: 488 nm; emission: 520 nm; Infinite M200, Tecan); serially diluted FITC-dextran was the standard (range: 0–12,000 μg/ml).

### Statistics

Statistical analyses were performed using GraphPad (GraphPad Software, San Diego, CA, United States). Survival curves analyses have been performed by Logrank test (Mantel Cox). For weight curves, a multiple unpaired *T*-test was performed per day with fewer assumptions corrected for multiple comparison with Holm–Sidak method. Normality and variance analysis were performed using Shapiro–Wilk normality test and one-way ANOVA (Brown-Forsythe test), respectively. For normal samples (Gaussian distribution) with equal variances two-way ANOVA has been performed to compare the effect of the strain and the dose for the first trial and of the sex and the dose for the second trial; multiple comparisons were carried out using Tukey’s test. For non-normal samples or/and with unequal variances non-parametric tests have been performed inside the groups (Kruskal–Wallis test); multiple comparisons were carried out using Dunn’s test. *P*-values less than 0.05 were considered statistically significant. More statistical details are included in each figure legend.

## Results and Discussion

### CD1 Mice Are Susceptible to DNBS-Induced Chronic Colitis

To design a murine model that will better predict results in humans, it is crucial to consider the real-life context of the target disease. IBD, including Crohn’s disease (CD) and ulcerative colitis (UC), are characterized by an abnormal activation of the gut immune system, which results in local chronic inflammation. Throughout their lives, patients with these diseases display active and inactive phases of variable duration that result in successive periods of relapse and quiescence. A good murine model must account for these disease dynamics. To trigger immune-mediated inflammation, it is possible to use haptenating agents, chemical compounds typically dissolved in ethanol. The ethanol allows the compounds to pass through the mucosal barrier. They then act upon either autologous or microbial proteins in the colon, rendering them immunogenic and thus provoking the abnormal activation of the immune system (Wirtz et al., 2007). As mentioned above, DNBS is one of the most common haptenating agents (Martin et al., 2017); it consistently induces chronic inflammation (Martin et al., 2014a). Most murine models of colitis use inbred strains, such as C57BL/6JRj (Black-6), and only employ males or females with the purported goal of limiting bias. However, this approach makes it problematic to transfer results to humans because representation of natural diversity in the mouse population is poor. Here, we wished to develop a more realistic murine colitis model, and we thus focused on three improvements to classical models: (i) mimicking the chronic nature of the disease; (ii) accounting for normal genetic variability by using outbred mice; and (iii) employing both female and male mice. More specifically, we used DNBS to chemically induce chronic inflammation in females and males of an outbred murine strain (RjOrl:SWISS [CD-1]) following the protocol described in **Figure 1**.

**FIGURE 2:**
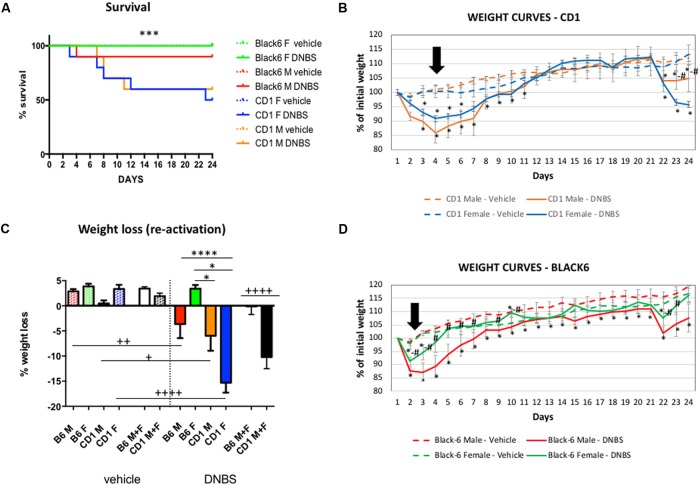
Survival rate **(A)** and body mass trends in CD-1 **(B)** and Black-6 **(D)** mice, and loss of body mass after second DNBS injection **(C)**. For the survival rate analysis Logrank test (Mantel Cox) was performed. For weight curves, a multiple unpaired *T*-test was performed per day with fewer assumptions corrected for multiple comparison with Holm–Sidak method, (^∗^) indicates significance vs. vehicle group and (#) significance between female and male individuals in DNBS treated groups. *n* = 10; *p* < 0.05. For the weight loss analyses, due to the lack of uniform variances when included the vehicle groups, two-way ANOVA was performed only in inflamed groups with strain and sex as factors followed by a Tukey test (results indicated as ^∗^). In order to compare the effect of the DNBS vs. the vehicle groups, a non-parametric Kruskal–Wallis test followed by a Dunn’s test was performed inside CD-1 and Black 6 groups separately (results indicated as +). *n* = 10; ^∗^*p* < 0.05, ^∗∗∗∗^*p* < 0.0001, ^+^*p* < 0.05, ^++^*p* < 0.01, ^++++^*p* < 0.0001. The black arrows indicate the moment when mice started to recover weight after the first DNBS injection. B6M, Black-6 males; B6F, Black-6 females; CD1M, CD-1 males; CD1F, CD-1 females; B6 M+F, Black-6 mice; CD-1 M+F, CD-1 mice.

In our first trial, we compared inflammation patterns in CD-1 and Black-6 mice; the latter is the inbred murine strain traditionally used in colitis models. We induced initial inflammation and then relapse by sequential injections of 200 mg/kg of DNBS; the dosage was thus mass calibrated. We observed that, although CD-1 mice were heavier than Black-6 mice (mean body mass: 29.9 g and 20.1 g, respectively), they were also more sensitive to inflammation in a significant way as observed in the survival curves (Log-rank test *p* = 0.0048). In fact, the mortality rate was 5% for Black-6 mice (0% for females and 10% for males) and 45% for CD-1 mice (50% for females and 40% for males) (**Figure 2A**). Survival curves analyses using Log-rank test also showed that the differences were also significant when sex differences were taken into account (*p* = 0.0368). Nevertheless, no statistical significant sex-related differences were found inside the different strains (*p* = 0.6793 and 0.3173 for CD-1 and Black-6 mice, respectively), supporting the accurateness of pooling female and male individuals. The pattern was the same when evaluating body mass (**Figures 2B–D**). CD-1 mice lost more body mass after the first and second injections than did Black-6 mice, being this effect more persistent during reactivation (**Figure 2C**). This effect was stronger in CD-1 females than in CD-1 males, indicating they are more sensitive to DNBS-induced colitis (**Figures 2B,C**). In Black-6 mice, the pattern was reversed: females lost less body mass than did males (**Figures 2C,D**). Two-way ANOVA analyses of inflamed mice showed the presence of strain effect (*p* = 0.0002) as well as interaction between sex and strain factors (*p* = 0.0021), confirming the differences observed. Furthermore, a delay at the beginning of the recovery period was observed in CD-1 mice, while Black-6 mice started to recover at days 2–3, CD-1 mice began at day 4 (**Figures 2B,D**).

**FIGURE 3:**
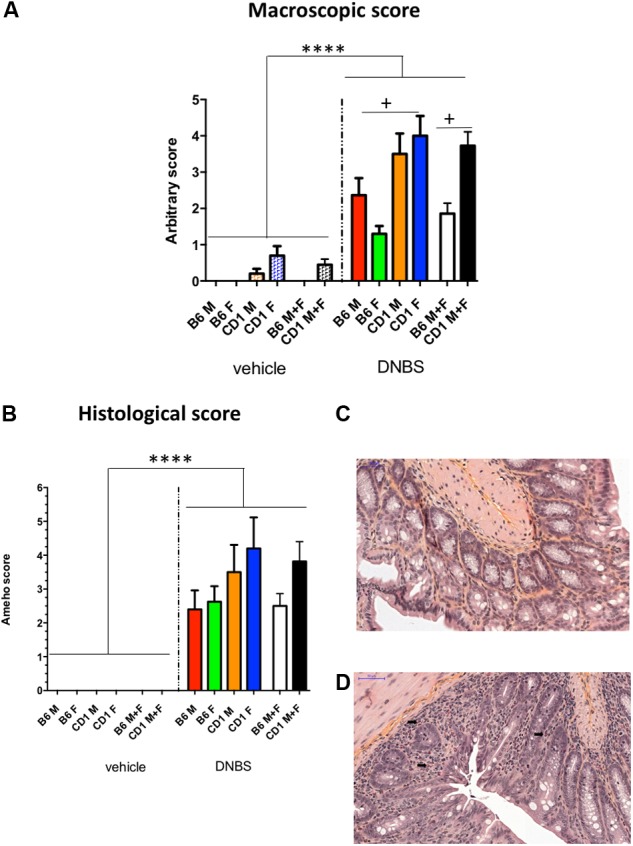
Macroscopic **(A)** and histological scores **(B)** and representative images of Black-6 mice **(C)** and CD-1 mice **(D)**. As both scores do not follow a Gaussian distribution, in order to compare the effect of the DNBS vs. the vehicle groups, a non-parametric Kruskal–Wallis test followed by a Dunn’s test was performed inside CD-1 and Black 6 groups separately (results indicated as ^∗^). The same test was performed for testing differences among inflamed groups (results indicated as +). *n* = 10; ^∗∗∗∗^*p* < 0.0001, ^+^*p* < 0.05. The black arrows indicate eosinophils. B6M, Black-6 males; B6F, Black-6 females; CD1M, CD-1 males; CD1F, CD-1 females; B6 M+F, Black-6 mice; CD-1 M+F, CD-1 mice.

Three days after the second DNBS injection, all the mice were sacrificed, and their colons were recovered for sampling and scoring. The macroscopic scores, which took into account the presence of ulcers, adhesions, hyperemia, altered transit, and colon wall thickness, provided complementary evidence that CD-1 mice were more sensitive than Black-6 mice to inflammation (**Figure 3A**). The sex-specific patterns in macroscopic scores mirrored those seen for body mass: CD-1 females had higher scores than did CD-1 males, indicating greater sensitivity, and Black-6 females had lower scores than did Black-6 males, indicating lesser sensitivity or a failure of colitis induction (see next paragraph). Histological scoring yielded similar results (**Figure 3B**). Of note, levels of eosinophils were higher in CD-1 mice than in Black-6 mice (**Figures 3C,D**).

Traditionally, the dosage of the haptenating substance is based on body mass. However, because Black-6 females and males differed dramatically in mass (mean body mass: 17.8 and 22.3 g, respectively), this approach may have been inappropriate. Black-6 females received lower doses of DNBS because of their lighter mass, and that dose might have been too low to trigger inflammation. However, it is difficult to conclude if the lack of inflammation was due to the low DNBS dose and/or to a possible difference in sensitivity between females and males. However, significant sex-specific differences in body mass were also observed in CD-1 mice (mean body mass for females and males: 27.6 and 32.2 g, respectively), and females were clearly more sensitive than males to inflammation. However, because standard deviation values were not very large, it was possible to pool females and males for most of the characteristics analyzed (**Figures 2C**, **3A,B**).

Ultimately, one of the goals of murine colitis models is to test the efficacy of candidate anti-inflammatory agents, including probiotics. Using the model described here, we would expect effective treatments to result in an improvement in inflammation-related symptoms. More specifically, mortality rates should decline, body mass should recover more quickly, and macroscopic and histological scores should be lower. The degree of improvement would be proportional to agent efficacy, but it would not necessarily reveal the mechanisms involved. An important caveat is that the underlying mechanism for DNBS-induced colitis is abnormal stimulation of the immune system. As a result, the model is not suitable for testing certain anti-inflammatory agents. For instance, probiotics, molecules or others that provide functional benefits unrelated to inflammation, such as excluding pathogens or modulating metabolic processes, could not be properly tested using this model.

### Chronic Colitis Severity in CD-1 Mice Can Be Modulated Using DNBS Dosage

Once we had verified that CD-1 mice were good candidates for developing a murine model of chronic colitis, we performed a second experiment in which we modulated DNBS dose to obtain different degrees of colitis severity. This experiment allowed us to better characterize the model and to gather data that, in future studies, will clarify the appropriate DNBS dosage depending on agent type, presumed agent efficacy, and target disease. We tested three different doses of DNBS—1.5, 2.5, and 3.5 mg per mouse. We selected these doses using the findings from comparative experiments with Black-6 mice in the first trial, where a dose of around 4 mg per mouse resulted in a high mortality rate. Furthermore, the doses were not calibrated for body mass because the results from the first experiment showed that smaller doses might not produce sufficient inflammation, and that there are probably sex-related differences in DNBS sensitivity.

**FIGURE 4:**
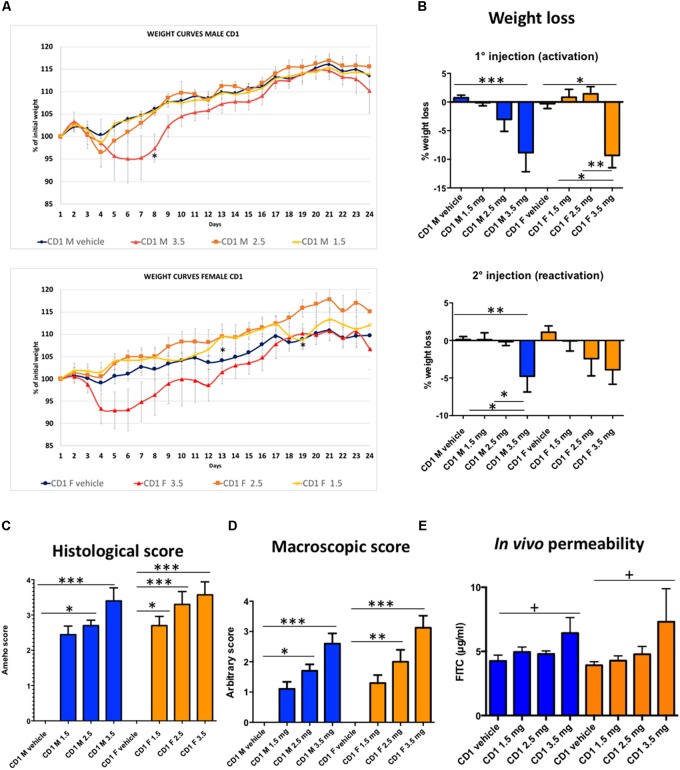
Response of CD-1 mice to colitis induced by different doses of DNBS. Body mass trends **(A)**, loss of body mass after the first DNBS injection and the second DNBS injection **(B)**, macroscopic **(C)** and histological scores **(D)** and *in vivo* permeability **(E)**. For weight curves, a multiple unpaired *T*-test was performed per day with fewer assumptions corrected for multiple comparison with Holm–Sidak method, (^∗^) indicates significance vs. vehicle group. *n* = 10; *p* < 0.05. No Gaussian data comparisons (loss of body mass and macro and histological scores) were performed using a non-parametric Kruskal–Wallis test followed by a Dunn’s test (results indicated as ^∗^). For the permeability analyses, two-way ANOVA was performed with dose and sex as factors followed by a Tukey test (results indicated as +). CD-1 males (M, in blue) and CD-1 females (F, in orange). *n* = 10. ^∗^*p* < 0.05, ^∗∗^*p* < 0.01, ^∗∗∗^*p* < 0.001, ^+^*p* < 0.05.

In the second experiment, mortality rates were lower: only two female mice, given a dose of 3.5 mg, died. As expected, after both DNBS injections, a dose-dependent effect on body mass was observed (**Figures 4A,B**). It is worth noting that loss of body mass was similar in females and males given the same dose. Taken together, these results suggest that CD-1 females are more sensitive to severe and severe-to-moderate inflammation (50% mortality at 4 mg of DNBS and 20% mortality at 3.5 mg); however, this sensitivity was not manifest when inflammation was moderate or low. A similar dose-dependent effect was seen in the macroscopic and histological scores (**Figures 4C,D**). Furthermore, the observed standard deviations were small, indicating that both sexes could be pooled in analyses.

### DNBS-Induced Chronic Colitis in CD-1 Mice Modifies Intestinal Permeability, Colon Cytokine Levels, and Lymphocyte Populations in the Spleen and Mesenteric Lymph Nodes (MLN)

As mentioned above, this murine model can be useful in two ways. First, the model’s general metrics such as loss of body mass, macroscopic scores, and histological scores could reveal the efficacy of potential treatments (e.g., anti-inflammatory compounds or probiotic strains). Second, the model could also help decipher the mechanisms underlying any positive effects. Because our model induces colitis using DNBS, it is best suited for examining immunomodulatory properties. For example, DNBS-provoked inflammation in Black-6 mice appears to arise from such mechanisms as altered gut barrier permeability and the activation of specific immune responses (Martin et al., 2014a). Consequently, this model could be used by researchers to study the specific effects of candidate anti-inflammatory agents on gut permeability and the immune system. Nevertheless, it is not possible to describe the expected results to be obtained when testing an anti-inflammatory agent as they will depend on their mechanisms of action.

Such permeability alterations are also present in CD-1 mice with DNBS-induced colitis (**Figure 4E**). Dysfunction of the epithelial barrier is a hallmark of inflammatory intestinal diseases. GIT permeability can be characterized by orally administering the paracellular tracer FITC-dextran. This technique reveals the degree of colon permeability and has been successfully linked to directly measure alterations in local permeability in colon tissues employing Ussing chambers (Martin et al., 2015). In this study, GIT permeability was modified in CD-1 mice challenged with different doses of DNBS (trial 2). Two-way ANOVA analysis showed that there is a dose effect (*p* = 0.0270), although no sex effect or interaction between both factors have been found (*p* = 0.9628 and *p* = 0.8642, respectively). Even if there was a clear response in males and females at the highest DNBS concentration tested (*p* < 0.005), basal permeability appears to be high, necessitating a strong dose of DNBS to obtain results (**Figure 4E**). These findings suggest that it may be problematic to use CD-1 mice to characterize permeability using moderate or low-grade inflammation models. However, we must interpret the results with caution since there is the possibility that permeability might be altered at other points along the GIT.

**FIGURE 5:**
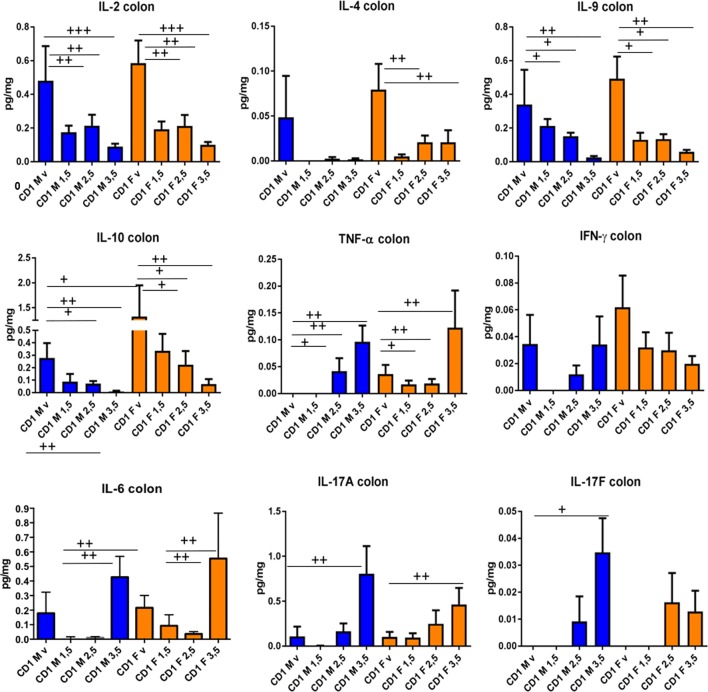
Colon levels of cytokines induced by different doses of DNBS in CD-1 mice. Analyses were performed by two-way ANOVA with dose and sex as factors followed by a Tukey test. CD-1 males (M, in blue) and CD-1 females (F, in orange) injected with vehicle (v), 1.5 mg (1.5), 2.5 mg (2.5), or 3.5 mg (3.5) of DNBS. *n* = 10; ^+^*p* < 0.05, ^++^*p* < 0.01, ^+++^*p* < 0.001.

**FIGURE 6:**
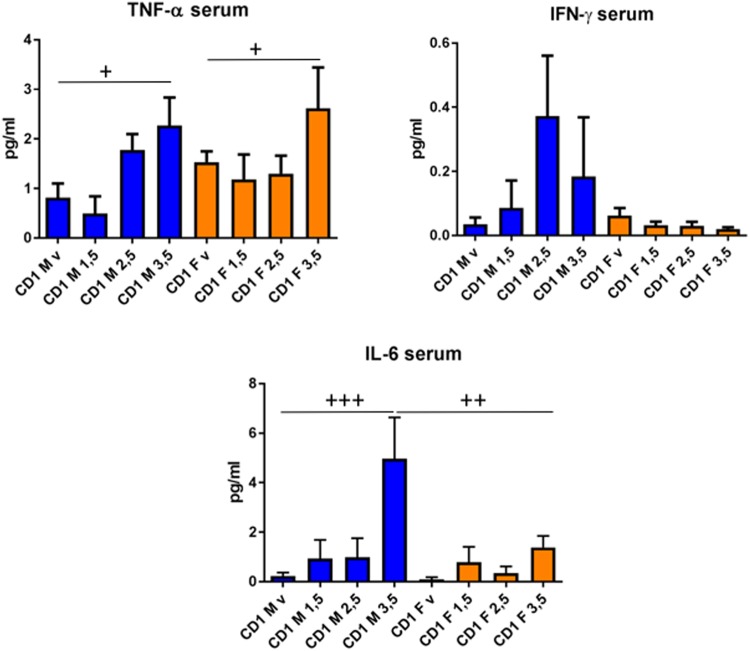
Serum levels of cytokines induced by different doses of DNBS in CD-1 mice. Analyses were performed by two-way ANOVA with dose and sex as factors followed by a Tukey test. CD-1 males (M, in blue) and CD-1 females (F, in orange) injected with vehicle (v), 1.5 mg (1.5), 2.5 mg (2.5), or 3.5 mg (3.5) of DNBS. *n* = 10; ^+^*p* < 0.05, ^++^*p* < 0.01, ^+++^*p* < 0.001.

It is apparent that DNBS-induced chronic colitis changes levels of several cytokines in CD-1 mice (**Figures 5**, **6**), confirming the generally dose-dependent nature of the inflammation response. We observed IL-9, IL-10, IL-17A, TNF-α, IL-2, IL-17F, IL-6, and IL-4 changes in the colon samples (**Figure 5**) and TNF-α and IL-6 in the serum samples (**Figure 6**). Two-way ANOVA analyses revealed dose-dependent responses for IL-2, IL-9, IL-10, TNF-α IL-6, IL-17A, and IL-17-B and sex influence on IL-4, IL-10, and serum IL-6 (*p* < 0.005). IL-10 is an important immunoregulatory cytokine—it reduces inflammation by suppressing the exaggerated mucosal immune response in the colon (Schreiber et al., 2000) and thus preserves the mucus barrier (Hasnain et al., 2013). It is a cytokine of reference in almost all murine colitis models. Similarly, we observed a decline in IL-9, IL-2, and IL-4. IL-9 controls intestinal barrier function (Gerlach et al., 2015); IL-2 is a potent inducer of T-cell proliferation and drives T-helper 1 (Th1) and Th2 effector T-cell differentiation (Hoyer et al., 2008); and IL-4 has anti-inflammatory properties. Indeed, IL-4 has the ability to stimulate alternative macrophages (M2s). In contrast to classical macrophages (M1s), M2s participate in a T helper type 1 (Th1)-polarized response and enhance the production of pro-inflammatory cytokines, which counteracts inflammation and promotes tissue repair (Goerdt et al., 1999; Gordon, 2003; Mantovani et al., 2007). In this sense, neutrophil activation, measured by myeloperoxidase (MPO) activity, reveal a week activation of neutrophils as MPO activity was similar for CD-1 mice that those in Black-6 mice treated with low doses of DNBS [(Martin et al., 2015) and data not shown]. As neutrophils are involved in inflammation, macrophage recruitment and M2s differentiation this result point also for a slight Th1 response in CD-1 mice.

**FIGURE 7:**
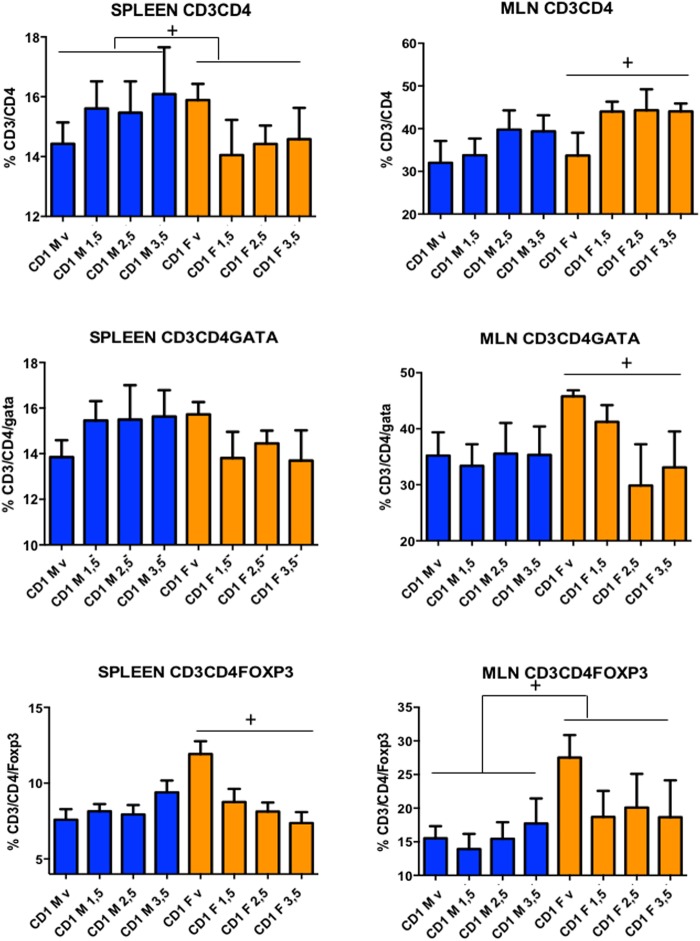
Levels of lymphocytes induced by different doses of DNBS in CD-1 mice. Lymphocyte populations were characterized using flow cytometry. Analyses were performed by two-way ANOVA with dose and sex as factors followed by a Tukey test. CD-1 males (M, in blue) and CD-1 females (F, in orange) injected with vehicle (v), 1.5 mg (1.5), 2.5 mg (2.5), or 3.5 mg (3.5) of DNBS. *n* = 10; ^+^*p* < 0.05, ^++^*p* < 0.01, ^+++^*p* < 0.001.

On the other hand, we saw an increase in IL-17A, IL-17F, IL-6, and TNF-α, underscoring that pro-inflammatory responses were occurring as well (Gabay, 2006; Bradley, 2008; Jin and Dong, 2013). The results for IFN-γ highlight that mouse strain matters: IFN-γ is a pro-inflammatory cytokine that plays a central role in DNBS-induced inflammation in Black-6 mice (Martin et al., 2014a), but that seemed to have the opposite effect in CD-1 mice (**Figures 5**, **6**). Consequently, it appears that DNBS and TNBS can elicit a Th1-mediated immune response (Randhawa et al., 2014) but that model functionality may vary depending on the host species and its genetic background (Mizoguchi and Mizoguchi, 2008; Mizoguchi, 2012). For instance, when treated with these compounds, SJL/J mice displayed a major Th1-mediated response (Neurath et al., 1995, 1996), while IFN-γ^-/-^ mice with a Balb/c background showed a Th2-mediated response (Dohi et al., 1999). In our study, to identify the major Th cell lines involved in the response of the CD-1 mice, T-cells from the spleen and MLNs were isolated and analyzed using flow cytometry. Several differences were found between male and female mice (**Figure 7**). CD-1 males had a weak response—there was a slight increase in CD3/CD4 cells in both the spleen and MLNs. CD-1 females had a different, stronger response—CD3/CD4 cells decreased in the spleen and increased in the MLNs (**Figure 7**). Furthermore, CD-1 females had diminished Th2 and Treg levels in both the spleen and MLNs (revealed by GATA-3 and Fox-p3 staining, respectively). In contrast, males displayed slightly increased Th2 levels in the spleen alone (**Figure 7**). These results, taken in tandem with the high levels of eosinophils (**Figure 3**) suggest that the Th2 response played a major role in CD-1 mice. The Th1 response, measured using T-bet staining, was not strong enough to be detected (data not shown). These findings indicate that, in future studies, it may be better to use females when testing for immunomodulation by candidate probiotics *in vivo*, especially if *in vitro* trials indicate that the mechanism of action involves changes in IL-10 production; IL-10 is produced by Treg cells, among others. However, it is necessary to broadly examine cytokine production and lymphocyte levels to fully clarify the mechanisms of action of any anti-inflammatory agent.

Overall, our findings allow us to recommend this model to test anti-inflammatory agents, including probiotics. We strongly recommend to perform the experiment at least in duplicate with a minimum of 10 mice per group. Nevertheless, this is a minimum, as the efficacy of the agent tested will determine the number of mice required to have statistical significant results. The use of 3.5 mg seems the better choice, however, as the dose-effect observed is usually animal facility-dependent, a preliminary study to optimize the dose is mandatory.

## Final Remarks

Here, we describe a murine model of chronic colitis in which inflammation was induced by the intrarectal administration of DNBS; it is novel because it used an outbred murine strain, CD-1, and employed both female and male mice. Ultimately, we want to make this model available to researchers who are testing the efficacy of anti-inflammatory agents, including probiotics (mainly NGPs), with immunomodulatory properties. The model could also serve to identify the anti-inflammatory agent potential mechanisms of action. Indeed, our aim is to provide the scientific community with a realistic alternative model for testing the efficacy of anti-inflammatory agents, for instance candidate probiotics—a model that can be customized based on agent type and target disease. We showed that it is possible to use an outbred murine strain without having any problems of reproducibility and that females and males can be pooled. Taken together, they yielded more representative results for some characteristics. However, combining results for the two sexes should be done with caution because we observed evidence of sex-specific sensitivity to severe inflammation protocols and sex-specific differences in some of the characteristics measured.

## Author Contributions

MB, LB-H, PB, PL, and RM designed the project. MB, FC, and RM designed and performed the experiments. MB, HS, and RM analyzed the results. MB and RM drafted the manuscript. MB, FC, HS, LB-H, PB, PL, and RM reviewed and edited the manuscript. All the authors read and approved the submitted version of the manuscript.

## Conflict of Interest Statement

The authors declare that the research was conducted in the absence of any commercial or financial relationships that could be construed as a potential conflict of interest.

## Supplementary Material

FIGURE S1 Our experimental design **(A)** in contrast to other acute models **(B,C)**. TNBS/DNBS **(B)** and DSS **(C)** classical protocols. In contrast to classical models of acute colitis with or without recovery, in our experimental design we have performed a chronic model in which a reactivation phase is included after recovery for better mimicking colitis flares and relapses.

FIGURE S2 Macroscopic score description. Representative figure of some of the symptoms: adhesion **(A)**, altered transit **(B)**, hyperemia **(C)**, and ulcers and thickening of colon wall **(D,E)**.
