# Supplementary figures and images for: A Versatile New Model of Chemically Induced Chronic Colitis Using an Outbred Murine Strain
Source: Front Microbiol. 2018 Mar 27;9:565. doi: 10.3389/fmicb.2018.00565 (PMC5881104; doi:10.3389/fmicb.2018.00565)

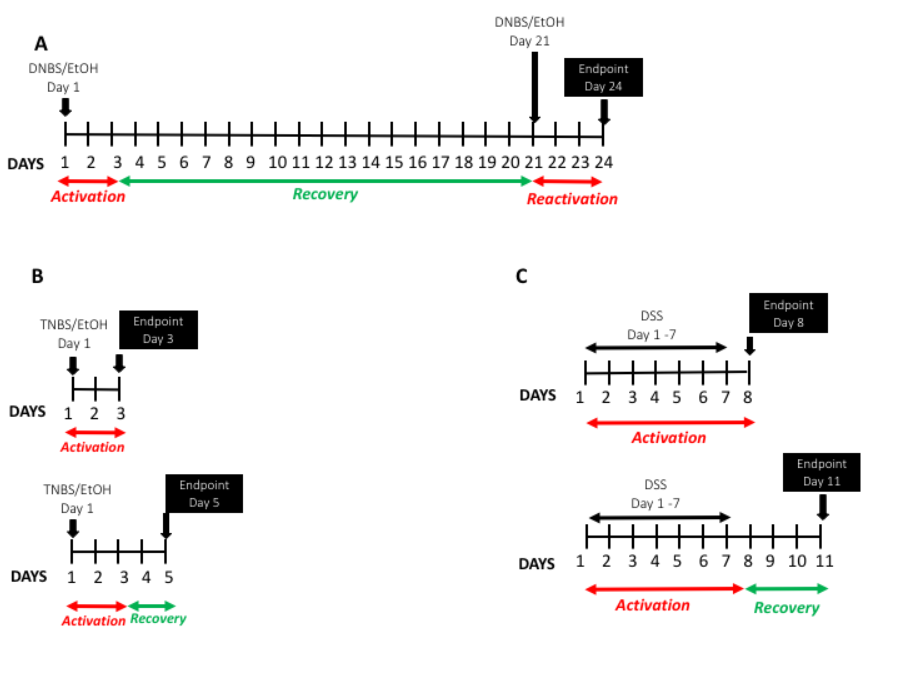

Supplement: FIGURE S1 — Our experimental design (A) in contrast to other acute models (B,C). TNBS/DNBS (B) and DSS (C) classical protocols. In contrast to classical models of acute colitis with or without recovery, in our experimental design we have performed a chronic model in which a reactivation phase is included after recovery for better mimicking colitis flares and relapses. [file Image_1.TIFF]

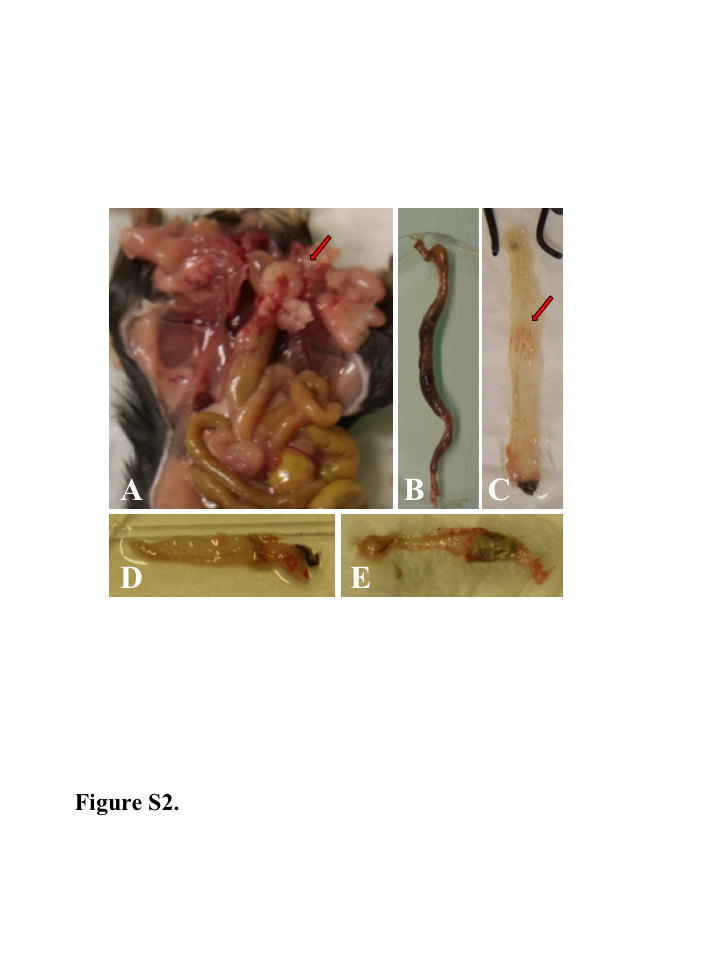

Supplement: FIGURE S2 — Macroscopic score description. Representative figure of some of the symptoms: adhesion (A), altered transit (B), hyperemia (C), and ulcers and thickening of colon wall (D,E). [file Image_2.TIFF]
